# Evaluation of the Central Effects of Systemic Lentiviral-Mediated Leptin Delivery in Streptozotocin-Induced Diabetic Rats

**DOI:** 10.3390/ijms222413197

**Published:** 2021-12-07

**Authors:** Kimberly A. Clark, Andrew C. Shin, Madhu P. Sirivelu, Ramya C. MohanKumar, Sreenivasa R. Maddineni, Ramesh Ramachandran, Puliyur S. MohanKumar, Sheba M. J. MohanKumar

**Affiliations:** 1 Neuroscience Graduate Program, Michigan State University, E. Lansing, MI 48824, USA; clarkki2@gmail.com (K.A.C.); psmohan@uga.edu (P.S.M.); 2 Neurobiology of Nutrition Laboratory, Department of Nutritional Sciences, College of Human Sciences, Texas Tech University, Lubbock, TX 79409, USA; Andrew.shin@ttu.edu; 3 Pathobiology and Diagnostic Investigation, Michigan State University, E. Lansing, MI 48824, USA; msp@pfizer.com; 4 Neuroendocrine Research Laboratory, University of Georgia, Athens, GA 30602, USA; rmohanku@sgu.edu; 5 Department of Poultry Science, College of Agricultural Sciences, The Pennsylvania State University, University Park, PA 16802, USA; srm265@gmail.com (S.R.M.); rameshr@psu.edu (R.R.); 6 Department of Biomedical Sciences, University of Georgia, Athens, GA 30602, USA

**Keywords:** diabetes, leptin, stress, hypothalamus, norepinephrine, corticosterone, gene transfer

## Abstract

Type 1 diabetes (T1D) is characterized by hyperphagia, hyperglycemia and activation of the hypothalamic–pituitary–adrenal (HPA) axis. We have reported previously that daily leptin injections help to alleviate these symptoms. Therefore, we hypothesized that leptin gene therapy could help to normalize the neuroendocrine dysfunction seen in T1D. Adult male Sprague Dawley rats were injected i.v. with a lentiviral vector containing the leptin gene or green fluorescent protein. Ten days later, they were injected with the vehicle or streptozotocin (STZ). HPA function was assessed by measuring norepinephrine (NE) levels in the paraventricular nucleus (PVN) and serum corticosterone (CS). Treatment with the leptin lentiviral vector (Lepvv) increased leptin and insulin levels in non-diabetic rats, but not in diabetic animals. There was a significant reduction in blood glucose levels in diabetic rats due to Lepvv treatment. Both NE levels in the PVN and serum CS were reduced in diabetic rats treated with Lepvv. Results from this study provide evidence that leptin gene therapy in STZ-induced diabetic rats was able to partially normalize some of the neuroendocrine abnormalities, but studies with higher doses of the Lepvv are needed to develop this into a viable option for treating T1D.

## 1. Introduction

The American Diabetes Association estimates that there are more than 30 million people in the U.S. currently living with diabetes. Approximately 1.25 million of these are Type I, or insulin-dependent diabetes mellitus (IDDM) patients. Type I diabetes (T1D) is thought to result from either an autoimmune disorder that results in the destruction of pancreatic beta cells that secrete the hormone insulin or from unknown causes resulting in loss of pancreatic beta cell function [1,2,3]. Many complications arise from the disease including cardiovascular dysfunction, kidney damage, retinopathy and peripheral neuropathy [2,3,4,5,6,7]. In addition, a number of central changes such as hyperphagia, polydipsia, hyperglycemia and activation of the hypothalamic–pituitary–adrenal (HPA) axis also occur in uncontrolled T1D [8,9,10]. Leptin is likely to be involved in the central dysregulation observed in T1D, since leptin levels are markedly reduced in T1D [11,12,13] and leptin regulates neuronal systems that control feeding and HPA function [10,14,15].

The reduction in leptin levels is probably the main contributing factor for the hyperphagia, polydipsia and HPA activation that is observed in T1D. This is because leptin can reduce food intake [16,17], alter sympathetic outflow, increase energy expenditure and thermogenesis [18,19] and suppress HPA activity [15,20,21,22,23] in non-diabetic animals. Therefore, it is logical to expect that the reduction in leptin levels in T1D contribute to hyperphagia and increased HPA activity. This led us to postulate that treating diabetic animals with leptin would ameliorate many of the diabetes-induced central and neuroendocrine dysfunction. In a previous study, we treated diabetic rats with daily injections of leptin (100 µg/kg BW/day s.c.) and this was able to partially reverse some of the effects of T1D, although it did not increase serum leptin levels to the levels observed in non-diabetic controls [10]. Another study used a higher dose of leptin (4 mg/kg BW/day) administered using minipumps and found that it was capable of restoring euglycemia in diabetic rats [24]. This prompted us to hypothesize that if leptin levels can be normalized in diabetic rats, it is possible that many of the central and neuroendocrine effects observed in T1D can be reversed.

A number of studies have used viral transfection of leptin through i.c.v. administration in an attempt to increase leptin levels and decrease obesity [25,26,27,28]. Dhillon et al. demonstrated that central administration of recombinant adeno-associated viral vector (rAAV) containing leptin cDNA to normal rats reduces age-related weight gain, adiposity and serum insulin levels [29]. Muzzin et al. have demonstrated that a single i.c.v. injection of rAAV-lep reduced body weight and food intake in both normal and Zucker *fa/fa* rats [30]. Several studies have used leptin gene transfer to treat diet-induced and age-related obesity and non-insulin-dependent diabetes mellitus (NIDDM) using animal models [25,31,32]; however, little work has been carried out in insulin-dependent diabetes mellitus and neuroendocrine alterations. Additionally, many of these studies involve central leptin gene therapy that can be difficult to translate to clinical settings. In the current study, we used a lentiviral vector, based on the human immunodeficiency virus-1 (HIV-1), a form of retrovirus that can infect both dividing and non-dividing cells and can be administered systemically for consistent gene expression [33]. We attempted systemic leptin lentiviral (Lepvv) transfection therapy and hypothesized that this will raise serum leptin levels in STZ-induced diabetic rats sufficiently to normalize the neuroendocrine dysfunction seen in this disease.

## 2. Results

### 2.1. Food Intake

**Figure 1:**
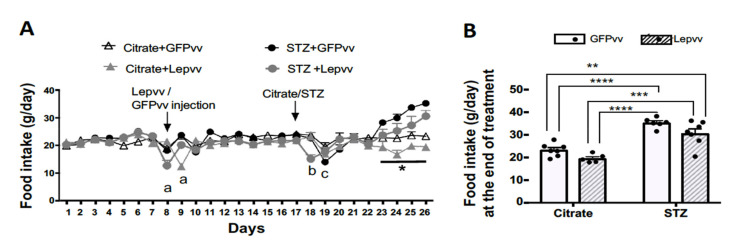
Food intake changes in male rats that were injected with Lepvv or GFPvv on day 8 followed by injection with citrate buffer or STZ (to induced diabetes) on day 17. (**A**) Profile of food intake (g/day, mean ± SEM) in diabetic and non-diabetic rats treated with Lepvv or GFPvv. Citrate + GFPvv (*n* = 7); STZ + GFPvv (*n* = 6); Citrate + Lepvv (*n* = 5) and STZ + Lepvv (*n* = 7) are represented. Data were analyzed using linear mixed models as described under Statistics. “a” indicates significant difference (*p* < 0.01) between Lepvv-injected rats and the other groups on days 8–9, “b” indicates significant difference (*p* < 0.0001) between diabetic rats injected with Lepvv and the other groups on day 18 and “c” indicates difference between GFPvv-injected diabetic and non-diabetic rats on day 19 (*p* < 0.01). * indicates *p* < 0.05 between Lepvv + citrate group and the other groups. (**B**) Average food intake on the 9th day after STZ/citrate injection (g, mean ± SEM). Data were analyzed by 2-way ANOVA followed by Tukey’s multiple comparisons test. Food intake was significantly reduced in the GFPvv-treated diabetic group compared to the citrate groups (**** *p* < 0.0001). Food intake in the Lepvv-treated diabetic animals was also increased compared to the citrate groups, but to a lesser extent (** *p* < 0.01; *** *p* < 0.001).

Changes in food intake (mean ± SEM; g) over the entire observation period in all the treatment groups are shown in Figure 1A. There were no significant differences between groups during the pre-treatment period (days 1–7). However, there was a drop in food intake immediately after Lepvv injection on day 8 and after STZ injection in the vector-injected groups. Most of the differences in food intake between groups occurred after Citrate/STZ injection on day 17. Food intake in the GFPvv + citrate group remained fairly stable until the end of the observation period. Food intake in the Lepvv + citrate treated group was markedly lower than that in the rest of the groups from day 23 to the end of observation. Overall, the diabetic groups consumed more food compared to the non-diabetic animals, indicating the development of hyperphagia. Figure 1B shows the average food intake (mean ± SEM; g) at the end of treatment. The average daily food intake for the diabetic groups was significantly higher than the non-diabetic groups (*p* < 0.001). While the food consumption in the GFPvv diabetic rat was dramatically different from the non-diabetic animals (*p* < 0.0001), food consumption in the Lepvv diabetic rat was less significant (*p* < 0.01 to *p* < 0.001 when compared to GFPvv + citrate and Lepvv + citrate groups, respectively).

### 2.2. Water Intake

**Figure 2:**
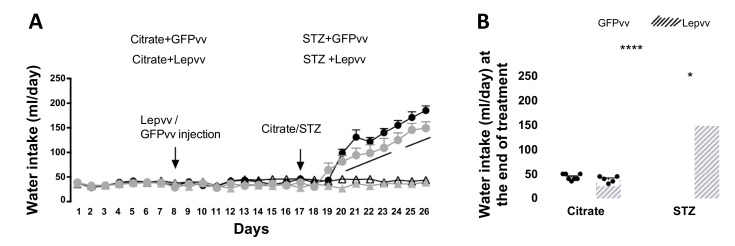
Water intake changes in male rats that were injected with Lepvv or GFPvv on day 8 followed by injection with citrate buffer or STZ (to induce diabetes) on day 17. (**A**) Profile of water intake (mL/day, mean ± SEM) in diabetic and non-diabetic rats treated with Lepvv or GFPvv. Citrate + GFPvv (*n* = 7); STZ + GFPvv (*n* = 6); Citrate + Lepvv (*n* = 5) and STZ + Lepvv (*n* = 7) are represented. Data were analyzed using linear mixed models as described under Statistics. “a” indicates significant difference between diabetic and non-diabetic groups (*p* < 0.0001). (**B**) Average water intake on the 9th day after STZ/citrate injection (g, mean ± SEM). Data were analyzed by 2-way ANOVA followed by Tukey’s multiple comparisons test. Diabetic animals treated with Lepvv had a modest reduction in water intake compared to GFPvv-treated animals (* *p* < 0.05). Diabetic groups had higher water intake than the non-diabetic groups. **** indicates *p* < 0.0001.

Changes in daily water intake (mean ± SEM; mL) over the entire observation period are shown in Figure 2A. There were no significant differences between groups during the pre-treatment period (days 1–7) and after lentiviral transfection (days 8–18). After the induction of diabetes, water intake began to increase rapidly in diabetic rats from day 20 and remained elevated compared to non-diabetic animals for the rest of the observation period (*p* < 0.0001). There was a modest reduction in diabetic animals treated with Lepvv only on day 21 (*p* < 0.05). Although the water intake appeared to be a little less in the Lepvv + STZ group compared to the GFPvv + STZ group for the rest of the observation period, this was not statistically different. Average water intake at the end of treatment in the diabetic groups was significantly higher than the non-diabetic groups (*p* < 0.0001; Figure 2B). The difference between the Lepvv + STZ group and the non-diabetic groups was less significant (*p* < 0.001) compared to the GFPvv + STZ group (*p* < 0.0001).

Moreover, water intake in the Lepvv + STZ group was modestly reduced compared to the GFPvv + STZ group (*p* < 0.05).

### 2.3. Body Weight

**Figure 3:**
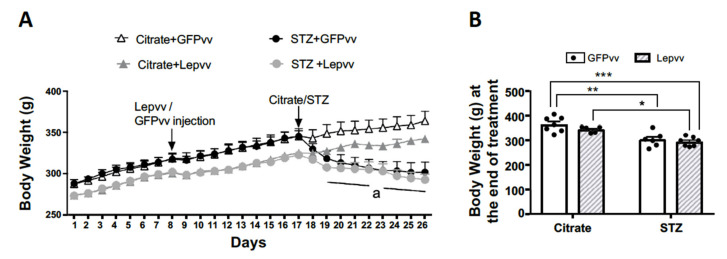
Body weight changes in male rats that were injected with Lepvv or GFPvv on day 8 followed by injection with citrate buffer or STZ (to induced diabetes) on day 17. (**A**) Profile of body weight (g, mean ± SEM) in diabetic and non-diabetic rats treated with Lepvv or GFPvv. Citrate + GFPvv (*n* = 7); STZ + GFPvv (*n* = 6); Citrate + Lepvv (*n* = 5) and STZ + Lepvv (*n* = 7) are represented. Data were analyzed using linear mixed models as described under Statistics. “a” indicates significant difference between diabetic rats that received Lepvv and non-diabetic rats that received GFPvv (*p* < 0.0001). Differences between diabetic and non-diabetic animals progressively increased towards the end of observation (*p* < 0.05–*p* < 0.0001). (**B**) Average body weight on the 9th day after STZ/citrate injection (g, mean ± SEM). Data were analyzed by 2-way ANOVA followed by Tukey’s multiple comparisons test. Diabetic rats lost more weight compared to the GFPvv treated non-diabetic group. Lepvv treatment caused a dramatic reduction in body weight in diabetic animals, which was still less when compared to non-diabetic rats treated with Lepvv. * indicates *p* < 0.05; ** *p* < 0.01 and *** *p* < 0.001.

Figure 3A shows the change in daily body weight (mean ± SEM; g) for all the treatment groups over the entire period of observation. Lepvv-treated rats weighed about 15 g less on average than GFPvv-treated rats but were not statistically different from each other. After vector transfection, the difference increased to ~24 g and was still not statistically different. There was no difference in body weight between the non-diabetic groups over time. In contrast, the diabetic groups lost a significant amount of weight. GFPvv-treated diabetic rats lost 42 g of weight on average, while Lepvv-treated rats lost only 31 g, although there was no statistical difference between these two groups. Figure 3B shows average body weights in the four groups at the end of treatment. While both diabetic groups lost weight compared to non-diabetic groups, the loss appeared to be more marked in the Lepvv + STZ groups (*p* < 0.001 compared to GFPvv + citrate and *p* < 0.01 compared to Lepvv + citrate). Body weight in the GFPvv + STZ group was significantly different from the GFPvv + citrate group only (*p* < 0.01).

### 2.4. Serum Leptin and Insulin and Blood Glucose

**Figure 4:**
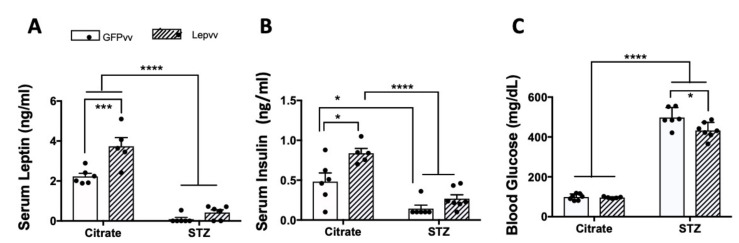
Changes in serum leptin (panel (**A**)), serum insulin (panel (**B**)) and blood glucose levels (panel (**C**)) in male rats that were injected with Lepvv or GFPvv followed by injection with citrate buffer or STZ (to induce diabetes). Citrate + GFPvv (*n* = 7); STZ + GFPvv (*n* = 6); Citrate + Lepvv (*n* = 5) and STZ + Lepvv (*n* = 7) are represented. Data were analyzed by 2-way ANOVA followed by Tukey’s multiple comparisons test. * indicates *p* < 0.05; *** indicates *p* < 0.001; **** indicates *p* < 0.0001.

Serum leptin levels (mean ± SEM; ng/mL) at the end of the treatment period are shown in Figure 4A. There was a significant effect of diabetes (*p* < 0.0001; F (1,19) = 150.4), vector treatment (*p* < 0.001; F (1,19) = 17.19) and interaction (*p* < 0.05; F (1,19) = 7.02). Serum leptin levels were significantly higher in non-diabetic rats treated with Lepvv (3.74 ± 0.4) compared to GFPvv-treated rats (2.23 ± 0.16; *p* < 0.001). Moreover, leptin concentrations in diabetic rats were significantly lower than non-diabetic animals, as expected (*p* < 0.0001). However, leptin concentrations remained similar in diabetic rats whether they were treated with GFPvv or Lepvv.

Lepvv transfection produced dramatic and modest increases in serum leptin and insulin levels in non-diabetic rats, respectively. Diabetic rats had significantly lower levels of leptin and insulin, but higher levels of blood glucose (*p* < 0.0001). Lepvv transfection in diabetic animals decreased blood glucose levels compared to GFPvv-treated rats (*p* < 0.05).

Serum insulin levels (mean ± SEM; ng/mL) at the end of the treatment period are shown in Figure 4B. There was a significant effect of diabetes (*p* < 0.0001; F (1,20) = 42.6) and vector treatment (*p* < 0.01; F (1,20) = 11.7). In non-diabetic rats, Lepvv transfection produced a modest increase in serum insulin levels (0.84 ± 0.06) compared to GFPvv transfected rats (0.48 ± 0.1; *p* < 0.05). These levels were four-fold higher than the levels in diabetic rats (0.14 ± 0.04 and 0.26 ± 0.04 in GFPvv and Lepvv groups, respectively; *p* < 0.0001). Insulin levels in non-diabetic GFPvv-treated rats were only three-fold higher than those in the diabetic GFPvv group (*p* < 0.05).

Mean blood glucose levels (mg/dL; Mean ± SEM) at the end of the treatment period are shown in Figure 4C. There was a significant effect of diabetes (*p* < 0.0001; F (1,21) = 728), vector treatment (*p* < 0.05; F (1,21) = 6.06) and interaction (*p* < 0.05; F (1,21) = 5.24). Glucose levels in diabetic animals were significantly higher than non-diabetic rats (*p* < 0.0001). Interestingly, Lepvv transfection significantly lowered blood glucose levels (432.86 ± 15) in diabetic animals compared to animals transfected with GFPvv (497.67 ± 20; *p* < 0.05).

### 2.5. HPA Axis Activity

#### Norepinephrine Concentrations in the PVN

**Figure 5:**
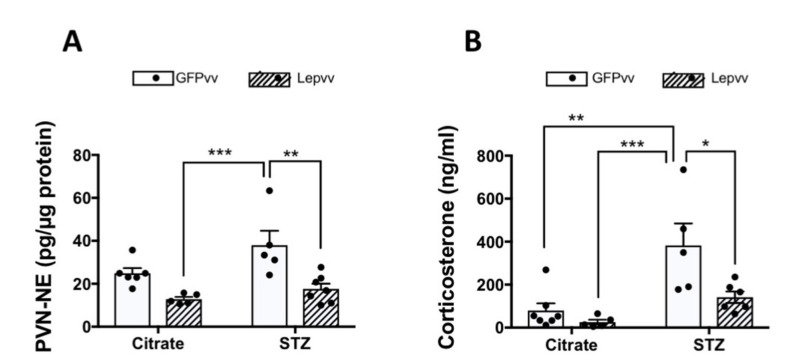
Effects of STZ-induced diabetes and leptin gene transfer on HPA axis activity. Citrate + GFPvv (*n* = 7); STZ + GFPvv (*n* = 6); Citrate + Lepvv (*n* = 5) and STZ + Lepvv (*n* = 7) are represented. Data were analyzed by 2-way ANOVA followed by Tukey’s multiple comparisons test. (**A**) Effects on NE concentrations in the PVN (pg/μg protein, mean ± SEM). Diabetic rats treated with GFPvv had higher concentrations of NE in the PVN than Lepvv-transfected rats (** indicates *p* < 0.01 and *** indicates *p* < 0.001). (**B**) Effects on serum corticosterone (ng/mL, mean ± SEM). Diabetic rats transfected with GFPvv had higher serum corticosterone levels compared to the rest of the groups. * indicates *p* < 0.05, ** indicates *p* < 0.01 and *** indicates *p* < 0.001.

NE concentrations (pg/µg protein; Mean ± SEM) were highest in the diabetic group transfected with GFPvv (37.9 ± 6.7; Figure 5A). Interestingly, Lepvv treatment significantly lowered NE concentrations in diabetic rats by half in the PVN (17.62 ± 2.4; *p* < 0.01). NE levels in the diabetic GFPvv-treated rats were also markedly higher than the non-diabetic Lepvv-treated rats (12.8 ± 1; *p* < 0.001). There were no differences in NE levels between non-diabetic animals.

### 2.6. Serum Corticosterone

Following NE levels in the PVN, serum corticosterone levels (ng/mL; Mean ± SEM) were highest in the diabetic group treated with GFPvv (383.3 ± 102; Figure 5B). These levels were significantly higher than the non-diabetic groups (*p* < 0.01). Interestingly, Lepvv transfection in diabetic rats significantly reduced corticosterone levels (141.3 ± 26; *p* < 0.05) compared to GFPvv-treated diabetic animals.

### 2.7. Vector and Leptin Expression in Adipose Tissue

**Figure 6:**
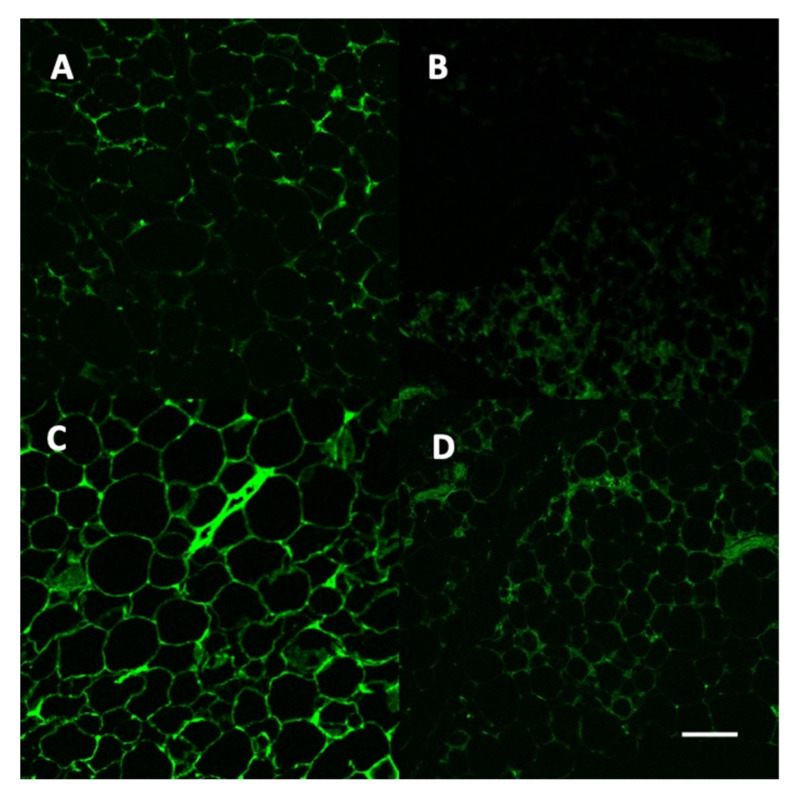
Representative images of adipose tissue from animals in the four groups using a confocal microscope; 20× objective. Panel (**A**): Citrate + GFPvv; Panel (**B**): STZ + GFPvv, Panel (**C**): Citrate + Lepvv; Panel (**D**): STZ + Lepvv. Green fluorescent protein indicates the presence of GFPvv in panels (**A**,**B**) and Lepvv in panels (**C**,**D**). Scale bar represents 200 µm.

Figure 6 demonstrates the presence of the vectors in the adipose tissue of the different treatment groups. Induction of diabetes appeared to reduce the presence of the vector in both GFPvv and Lepvv groups.

**Figure 7:**
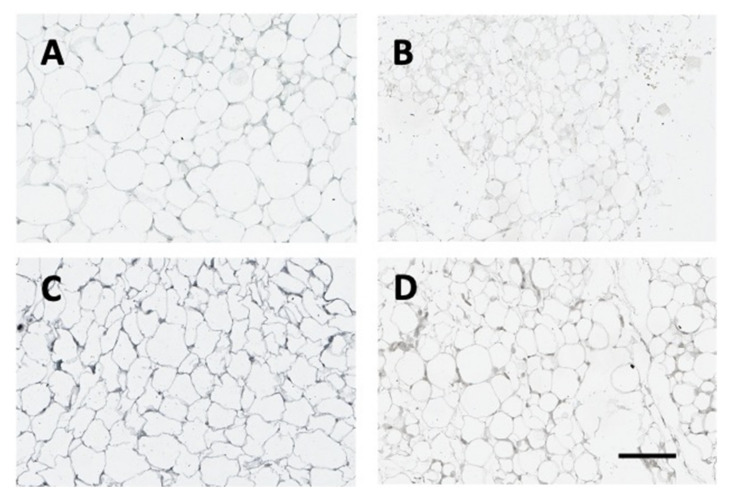
Representative images of adipose tissue from animals in the four groups. Tissue sections were subjected to leptin immunohistochemistry according to established protocols and scanned using an Aperio Digital slide scanner. Panel (**A**): Citrate + GFPvv; Panel (**B**): STZ + GFPvv, Panel (**C**): Citrate + Lepvv; Panel (**D**): STZ + Lepvv. Note the marked increase in leptin expression in the Lepvv group treated with citrate. Scale bar represents 200 µm.

Figure 7 (below) demonstrates leptin protein expression in adipose tissue in the different treatment groups. The strongest signal was apparent in the Lepvv + citrate group (Panel C). Treatment with STZ reduced the expression of leptin in both GFPvv and Lepvv groups.

## 3. Discussion

Results from the study indicate that GFPvv transfection in STZ-induced diabetic animals causes a significant increase in both food and water intake with a concomitant decrease in body weight. Lepvv transfection produced modest reductions in hyperphagia, polydipsia and body weight loss in the diabetic group towards the end of treatment. Interestingly, Lepvv transfection increased leptin and insulin levels in non-diabetic rats. More importantly, it reduced hyperglycemia and blocked the activation of the stress axis in diabetic rats.

Several studies have examined the effects of central leptin gene therapy using recombinant adenoviral vectors (rAAV-Lep) in obese [25,28,29,30,34,35] and normal rodents [26,27,29,36]. Only one study has used a lentiviral vector for leptin gene transfer, but this was administered centrally to study the effects on Alzheimer’s-like symptoms in presenilin mice [37]. Most studies have used leptin gene therapy to study its impact on body weight, food consumption and obesity. However, there are only a few studies that have examined the effect of leptin gene therapy in diabetic animals [35,38,39,40,41]. Of these, two studies used intravenous administration [35,41], two used central administration [38,39] and one used intramuscular administration of the viral vector [40]. The present study used a lentiviral vector for leptin gene therapy to treat type 1 diabetes for the first time. Moreover, the vector was administered systemically rather than centrally for added translational value. An earlier study that used an intramuscular injection of rAAV-lep produced circulating leptin levels of 2–5 ng/mL and was capable of reducing food intake, body weight, hyperglycemia and insulin levels in leptin-deficient ob/ob mice for a 6-month period [40]. However, we were unable to detect any changes in food intake after intramuscular injection of Lepvv (data not shown) in rats and resorted to i.v. injection of Lepvv. Tail vein injection of rADV-Lep in another study was capable of decreasing feeding in ob/ob mice and reduced body weight by 50% in 3 weeks. However, the animals resumed normal feeding and regained weight and reached 83% of control body weight by 17 weeks [35]. These studies demonstrate the effectiveness of systemic gene therapy; however, the duration of the effects observed could depend on the type of vector used.

Lepvv transfection in the present study was capable of significantly increasing serum leptin levels in non-diabetic but not in diabetic animals. In non-diabetic animals, leptin levels increased to 4 ng/mL after Lepvv treatment. Immunohistochemistry results indicate that Lepvv is capable of integrating into adipose tissue and expressing leptin. However, it was not sufficient to increase circulating leptin concentrations especially in diabetic animals. In another study, i.v. administration of ADVlep to STZ-treated diabetic rats resulted in a sharp increase in circulating leptin concentrations (over 200 ng/mL) a week after vector injection, which dropped sharply in 2 weeks and remained elevated (about 10 ng/mL) even at 30 days [41]. The reason for the lack of increase in leptin concentrations in diabetic animals in the present study is not clear. The type of vector used and the dose of the vector (our dose was 1000-fold less) could have been major contributing factors. The absence of a visible effect on serum leptin concentrations in diabetic animals could also be due to the substantial loss of body fat in these animals.

In contrast to its effect on leptin concentrations, transfection with Lepvv increased serum insulin concentrations in non-diabetic animals compared to GFPvv-treated non-diabetic rats. This is in contrast to other studies that have observed decreases in insulin concentrations after leptin gene therapy [26,29,35,40]. More importantly, Lepvv treatment partially normalized the reduction in insulin concentrations and hyperglycemia associated with T1D. This is evident in the fact that insulin concentrations in the Lepvv + STZ group were not different from the GFPvv + citrate group. This effect of Lepvv could be due to a direct stimulatory action on pancreatic islets, as demonstrated by Kojima et al. [38]. Yu et al. have also shown that there is a near significant increase in pancreatic ß cell abundance after systemic leptin gene therapy [41]. The other possibility is that Lepvv might reduce insulin clearance by decreasing the production or activity of CEACAM-1 in the liver [42]. The modest increase in insulin concentrations produced by Lepvv is corroborated by a modest reduction in hyperglycemia resulting from diabetes. The study by Yu et al. also shows that systemic administration of leptin adenoviral vector dramatically decreases blood glucose levels, 30 days after vector administration. While blood glucose levels begin to rise in these animals after 30 days, they still remain significantly low compared to untreated diabetic animals. The same study used NOD mice that were severely diabetic and systemic leptin gene therapy resulted in a remarkable improvement in hyperglycemia and ketosis and improved body weight for nearly 6 months [41]. Increase in insulin-like growth factor-1 expression and inhibition of gluconeogenesis were some of the mechanisms that were reported in that study. Other studies with rAAV-lep have found that a single icv injection can reverse hyperglycemia by increasing the rate of glucose disposal and improving insulin sensitivity in diabetic mice [39]. It was proposed that the central rAAV-lep treatment increases non-shivering thermogenesis in brown adipose tissue, leading to increased glucose usage and improved insulin sensitivity. This effect was independent of pancreatic insulin production [39]. Central rAAV-lep was also capable of reversing hyperglycemia and body weight loss in STZ-treated mice. It also improved their survival. Blood glucose levels in these animals were normalized by 8 weeks and remained low for the next 44 weeks [38].

The lack of pronounced effects on food intake, water intake and serum insulin and hyperglycemia in the present study could be attributed to a couple of factors. One was perhaps the lower dose of lentiviral vector particles used. Second, a duration of more than 10 days is probably necessary for gene expression induction by the viral particles. If this was followed by a longer period of observation, we could have had a better understanding of the effect of Lepvv transfection on the parameters mentioned above. Regardless, even during this short observation period, Lepvv transfection caused a significant reduction in NE concentrations in the PVN of diabetic animals. This effect could be mediated through a direct inhibitory effect of leptin on brainstem noradrenergic neurons [15], or indirectly through the stimulation of GABAergic neurons at the level of the hypothalamus [43]. The reduction in hypothalamic NE levels was accompanied by a marked reduction in circulating corticosterone in these rats. This reduction in HPA axis activity is of great importance as chronic hyperactivation of the stress axis is known to produce a myriad of deleterious effects, which may further compromise the health of diabetic patients [44].

In conclusion, results from the present study indicate that systemic administration of Lepvv to STZ-induced diabetic rats was able to partially normalize the central and neuroendocrine abnormalities studied. However, the effects need to be examined with higher doses of the vector and for a longer duration of observation to determine the effectiveness of the lentiviral leptin gene therapy. More third generation lentiviral vectors are currently being used in clinical practice [45]. Further investigation may help the utility of Lepvv as either an alternative or adjunctive treatment for T1D.

## 4. Materials and Methods

### 4.1. Animals

Adult male Sprague Dawley rats (3–4 months old) weighing approximately 350 g were obtained from Harlan Sprague Dawley Inc., Indianapolis, IN. They were housed in light-controlled (light on from 0700–1900 h), air-conditioned (23 ± 2 °C) animal quarters and were fed rat chow and water ad libitum. After a week of rest, their food intake, water intake and body weight were recorded daily for a period of 1 week, after which they were used in experiments as described below. Animals were used in the experiments in accordance with the NIH guide for the care and use of laboratory animals and were approved by the Institutional Animal Care and Use committee and the Institutional Biosafety committee at Michigan State University (Project # 06/07-099-00; approved 08/31/2007; IACUC-MSU). They were completed in accordance with the ARRIVE guidelines.

### 4.2. Plasmid Construction

A full-length (531 base pairs) rat leptin cDNA PCR product was amplified with forward (AGACGCGGATCCGCGATGTGCTGGAGACCCCTGT) and reverse primers (AGACGCGTCGACTCAGCATTCAGGGCTAAG) using a rat cDNA library as the template. The restriction enzyme sites that recognize BamH I and Sal I were added to forward and reverse primers, respectively, to facilitate subcloning. The rat leptin cDNA was then subcloned into a lentiviral vector plasmid that carries cytomegalovirus gene promoter and woodchuck hepatitis virus post-transcriptional element besides some of the lentiviral genome. The resultant plasmid (pRleptin.lenti) was utilized for the production of lentiviral particles to overexpress leptin.

### 4.3. Production of Leptin Lentiviral Particles

Human embryonic kidney 293T (HEK) cells (3 × 10^5^) were plated on 100 mm cell culture plates and transfected using calcium phosphate the following day with 3 plasmids: 10 µg of pRleptin.lenti or pGFPlenti (control plasmid that carries cDNA encoding green fluorescent protein (GFP)), and two other lentiviral packaging plasmids (3.5 µg of pVSV-G and 6.5 µg of pGag-Pol). Infected HEK cells were maintained in Dulbecco’s modified Eagle’s medium (DMEM) (Invitrogen, Carlsbad, CA, USA) supplemented with 10% fetal bovine serum (Gibco) and 1% antibiotic solution (10,000 IU/mL penicillin and 10,000 µg/mL streptomycin, Gibco) at 37 °C with 5% CO_2_. Conditioned medium was harvested 48 h and 72 h post-transfection, cleared of debris by centrifuging at low speed then filtered through 0.45 µm filters and stored at −80 °C. Concentrated vector stock was prepared by ultra-centrifugation of the supernatant at 20,000× *g* for 30 min using filter devices (Amicon Inc., Beverly, MA, USA) and stored at −80 °C. HEK cells were transduced with either rat leptin lentiviral particles or GFP lentiviral particles for evaluating the expression of leptin or GFP. The cell culture media collected from leptin or GFP-transduced HEK cells were also tested for leptin or GFP expression.

### 4.4. Experimental Design

After the first week of observation, animals were randomly divided into two groups and administered 80 µL of medium containing 3.2 × 10^9^ purified VP/mL of GFPvv or Lepvv via intravenous infusion in the jugular vein under isoflurane anesthesia. Ten days later (day 17), they were further divided into two groups each. One group was treated with the vehicle for streptozotocin (STZ) and the other with a 2% solution of Streptozotocin (STZ) (Sigma Chemical Co., St. Louis, MO, USA) in cold 0.1 M citrate buffer, pH 4.5 i.p.). Altogether, there were 4 groups: GFPvv + Citrate (*n* = 7), GFPvv + STZ (*n* = 6), LepLvv + Citrate (*n* = 5) and LepLvv + STZ (*n* = 7). The day following STZ treatment, blood from the tail vein was used to measure blood glucose levels using a glucometer (Accucheck, Boehringer-Mannheim, Indianapolis, IN, USA). Animals with blood glucose levels of 150 mg/dL or higher were considered diabetic.

Body weight, food and water intake were monitored daily for 9 days. At the end of the observation period, animals were sacrificed, and brains were quickly removed and frozen. Trunk blood was collected, and serum was assayed for leptin, corticosterone (CS) using RIA and insulin using ELISA.

### 4.5. RIA: Corticosterone and Leptin

Commercial RIA kits were used to measure serum levels of CS (Diagnostic Products Corp. Los Angeles, CA, USA) and leptin (Linco Research, St. Charles, MO, USA). The samples were assayed in duplicate according to the manufacturer’s instructions. The sensitivity of the CS assay was 0.2 ng/mL and that of leptin was 0.5 ng/mL. The intra-assay variability for CS was 5.74% and for leptin was 3.55%.

### 4.6. Insulin Assay

A commercial ELISA kit was used to measure serum levels of insulin (Linco Research, St. Charles, MO, USA). The samples were assayed in duplicate according to the manufacturer’s instructions. The sensitivity of the assay was 0.2 ng/mL.

### 4.7. Brain Microdissection

Serial sections (300 µm thick) of the brain were obtained using a cryostat maintained at −10 °C. The sections were transferred to cover slips, which were placed on a cold stage set at −10 °C. The sections between 1500 and 2100 µm posterior to the bregma were selected for paraventricular nucleus (PVN) microdissection. Using the stereotaxic atlas as a reference, the PVN was dissected bilaterally from the dorsal aspect of the third ventricle. Tissue samples included all subdivisions of the nuclei and were stored at −70 °C. At the time of analysis, tissue samples were homogenized in 0.05 M perchloric acid. After removing an aliquot for protein assay, the remaining sample was centrifuged, and the supernatant was used for HPLC analysis.

### 4.8. High-Performance Liquid Chromatography with Electrochemical Detection (HPLC-EC)

The HPLC-EC system consisted of an LC-4C amperometric detector (Bioanalytical Systems, West Lafayette, IN, USA), a phase II, 5 µm ODS reverse phase, C-18 column (Phenomenex, Torrance, CA, USA), a glassy carbon electrode (Bioanalytical Supplies, West Lafayette, IN, USA), a CTO-10 AT/VP column oven, and an LC-10 AT VP pump (Shimadzu, Columbia, MD, USA). The composition of the mobile phase was as follows: monochloroacetic acid (14.14 g/L), sodium hydroxide (4.675 g/L), octanesulfonic acid disodium salt (0.3 g/L), ethylenediaminetetraacetic acid (0.25 g/L), acetonitrile (3.5%), and tetrahydrofuran (1.4%). The mobile phase was made in pyrogen-free water and then filtered and degassed through a Milli-Q purification system (Millipore, Bedford, MA, USA) and pumped at a flow rate of 1.8 mL/min. The range of the detector was 1 nA full scale, and the potential of the working electrode was 0.65 V. At the time of HPLC analysis, the tissues were homogenized in 0.05 M HCLO4 and centrifuged at 12,000 rpm for 10 min. A mixture of 50 µL of the supernatant and 25 µL of the internal standard (0.5 mM dihydroxybenzamine) was injected into the HPLC system. Chromatograms were analyzed using the Chromatopac (v 6.4) software (Shimadzu, Columbia, MD, USA). The sensitivity of the HPLC system is less than 1 pg.

### 4.9. Confocal Microscopy and Leptin Immunohistochemistry

Tissues from animals in the different treatment groups were fixed in formalin, embedded in paraffin and sectioned at 4 µm. They were subjected to leptin immunohistochemistry using standard protocols that involved deparaffinization in xylene followed by dehydration in graded alcohol. Sections were permeabilized in 0.25% triton in PBS, subjected to endogenous peroxidase blocking and blocked for an hour using PBS-Casein blocking buffer. They were then incubated with leptin antibody tagged with HRP (1:200; Bioss BS0409R) overnight at room temperature. DAB with nickel enhancement was used for color development. The slides were scanned with an Aperio digital scanner for brightfield images and a Zeiss LM710 confocal microscope was used for fluorescent images.

### 4.10. Protein Assay

A 10 µL aliquot of the tissue homogenate was used in duplicate in a protein assay. Protein levels were determined using a microplate bicinchoninic acid assay (Pierce, Rockford, IL, USA). Absorbance at 562 nM was obtained using an ELX 800 microplate reader (Biotek Instruments, Winooski, VT, USA). Neurotransmitter concentrations were expressed as picogram per microgram protein.

### 4.11. Statistical Analysis

All analyses were performed using SAS V 9.4 (Cary, NC, USA) by Dr. Deborah Keys, our statistical consultant. A significance threshold of 0.05 was used. A two-way ANOVA was used to test for differences in glucose, insulin, leptin, corticosterone and norepinephrine concentrations due to vectors in diabetic and non-diabetic rats. Multiple comparisons were adjusted for using Tukey’s test.

Linear mixed models were used to analyze food and water intake and body weight for vector effects in diabetic and non-diabetic rats. Model residuals were examined to evaluate the assumption of normality. Satterthwaite’s degrees of freedom method were used. Random intercepts for each rat were used to account for within-rat correlation. The full linear mixed models included a fixed factor of vector and fixed covariate of time and a vector by time interaction. Separate models were used for diabetic and non-diabetic rats. Vector by time interaction effects were used to test if change in food or water intake or body weight over time was different between vectors.

## Data Availability

All data generated during the course of the studies are presented in this manuscript.
